# Intratumoral IL-15 potentiates radiation-induced anti-tumor immunity

**DOI:** 10.1186/2051-1426-3-S2-P239

**Published:** 2015-11-04

**Authors:** Karsten Pilones, Joseph Aryankalayil, Silvia Formenti, Sandra Demaria

**Affiliations:** 1 Weill Cornell Medical College, New York, NY, USA; 2 NYU School of Medicine, New York, NY, USA; 3 Weill Cornell Medical College, Radiation Oncology Department, New York, NY, USA; 4 NYU School of Medicine, Pathology Department, New York, NY, USA

## 

Radiotherapy (RT) can induce T cell-mediated anti-tumor immune responses by multiple mechanisms but is often unable to overcome immunosuppression in the tumor microenvironment. The common gamma-chain cytokines interleukin (IL)-2 and IL-15 promote the proliferation of activated T cells and, therefore, are prime agents for immunotherapy strategies aimed at sustaining anti-tumor T cell responses. The benefits of high dose IL-2, however, are undermined by serious toxicity and by regulatory T cell (Treg) stimulation. In contrast, IL-15 is well-tolerated and lacks Treg stimulatory activity, making it an attractive candidate for testing in combination with RT. Here we tested the hypothesis that IL-15 strengthens the pro-immunogenic effect of local RT to potentiate a durable anti-tumor immune response.

The poorly immunogenic mouse TSA breast cancer cells were implanted s.c. in syngeneic BALB/c mice and randomly assigned to one of 4 treatment groups when tumors reached 5mm average diameters: control, RT, IL-15 or RT+IL-15. RT was delivered locally in 8 Gy fractions on days 13, 14 and 15. IL-15 (2 μg/mouse) was administered s.c. peritumorally daily for 10 days starting on day 12. Mice were followed for tumor growth. A parallel experiment was done to characterize tumor-infiltrating lymphocytes (TILs) at the end of treatment (day 22).

Low dose IL-15 given peritumorally as a monotherapy induced marginal tumor growth control and had no effect on survival (median survival = 45 days compared to 76 days for control). Local RT significantly delayed tumor growth (p < 0.05 compared to control) and improved survival (median= 76 days, p < 0.05). However, highest survival advantage was seen in mice given IL-15+RT (median=102 days, p < 0.05 compared to all groups) with 1 of 6 mice showing complete tumor rejection and development of anamnestic response against tumor re-challenge. Analysis of TILs showed marked infiltration of CD8+ T cells expressing activation marker CD137 (35.3% in RT+IL-15 vs 5.9% in control, p < 0.05) while the increase was modest with either monotherapy (18.8% in RT, 20.7% in IL-15, p < 0.05 compared to control). In addition, we found a significant increase in the ratio of effector CD4+ T cells to Tregs (2.5 in RT+IL-15 versus 0.78 in control, p < 0.05) whereas monotherapy had no effect (1.14 in RT, 0.96 in IL-15).

Overall these results support the rational combination of low dose intratumoral IL-15 with local RT to re-awaken immunity against poorly immunogenic tumors. We are currently elucidating the mechanisms involved in pre-clinical models in preparation for future testing in patients.

